# A Novel Approach Against Male Pattern Hair Loss With Topical Dimethylglycine Sodium Salt (DMG‐Na) and Caffeine: Efficacy of a 24‐Week, Double‐Blind, Randomized, Placebo‐Controlled Trial

**DOI:** 10.1111/jocd.70390

**Published:** 2025-08-18

**Authors:** Leonardo Celleno, Carolina Bussoletti, Maria Vittoria Tolaini, Alfredo Rossi, Lorenzo Ala, Maike Becker, Jörn Michael Völker, Erik Schulze Zur Wiesche

**Affiliations:** ^1^ Product Testing Department Eurofins Biopharma Rome Italy; ^2^ Synlab Med srl. Firenze Italy; ^3^ Dermatology Clinic, Department of Clinical Internal. Anesthesiologic and Cardiovascular Sciences Sapienza University of Rome Rome Italy; ^4^ Dr. Kurt Wolff GmbH & Co. KG Bielefeld Germany; ^5^ Dr. August Wolff GmbH & Co. KG Arzneimittel Bielefeld Germany

**Keywords:** androgenetic alopecia, caffeine, dimethylglycine sodium salt, DMG, DMG‐Na salt, hair loss, male pattern hair loss, sodium dimethylglycinate, topical shampoo formulation

## Abstract

**Background:**

Male pattern hair loss is the most common form of hair loss in men, occurring in specific patterns. Contributing factors include hormonal changes, stress, malnutrition, and insufficient blood microcirculation.

**Objective:**

Topical applied dimethylglycine sodium salt (DMG‐Na) has been identified to increase skin microcirculation by activating endothelial nitric oxide synthase and inducing nitric oxide production. Given the importance of sufficient microcirculation on hair growth, the goal of this study was to evaluate the effectiveness of topically applied DMG‐Na in combination with caffeine against male pattern hair loss.

**Methods:**

A 24‐week, double‐blind, randomized, placebo‐controlled trial was conducted on 154 men with male pattern hair loss, treated with a DMG‐Na and caffeine‐containing shampoo or the corresponding placebo. The primary efficacy parameter was the change in the number of hairs pulled via hair pull test from baseline to 6 months of daily product application. Clinical efficacy was further evaluated via phototrichogram analysis on a subgroup of 30 subjects.

**Results:**

The decreased number of hairs pulled during the hair pull test after 6 months of active shampoo application was significantly higher compared to the placebo (−2.8 ± 1.6 vs. 0.6 ± 2.2; *p* < 0.001), with no reported adverse events. Phototrichogram results showed an increase in the number of hairs, hair density, and percentage of anagen hairs after 6 months of active shampoo usage (*p* < 0.001).

**Conclusion:**

The clinical efficacy of this novel DMG‐Na and caffeine‐containing shampoo demonstrates significant potential against male pattern hair loss, offering promising results without any undesirable side effects.

## 1 Introduction

Androgenetic alopecia, commonly known as male pattern hair loss, represents the most common type of hair loss in men, affecting almost every second man by the age of 50 years [1]. Its progression is characterized by well‐described patterns, beginning with a gradual thinning in the frontal and parietal regions, forming an M‐shaped recession, and subsequently advancing to hair loss at the vertex and the mid‐frontal scalp [1]. Although often perceived as a mild dermatological issue, its high incidence and the potential psychological burden (reduced self‐esteem, heightened anxiety, and depressive symptoms in certain individuals [2, 3]) highlight its considerable effect on overall quality of life. Despite the availability of various treatment options for men who choose therapy—including oral and topical approved medications, hormonal therapies, nutraceuticals, PRP (“platelet‐rich plasma”), microneedling, and more invasive techniques like hair transplantation—the treatment of AGA in men remains particularly challenging due to the inconsistent responses among patients to conventional therapies [4, 5]. Moreover, as the condition tends to persist and progress when treatment is discontinued, patients must adhere to a lifelong therapy [6].

Until today, topical minoxidil and oral finasteride are the only FDA‐approved drugs for the treatment of male pattern alopecia [1, 7]. However, due to a relatively low 30%–40% responder rate for topical minoxidil treatment in the overall population as well as the possible occurrence of adverse effects including itching of the scalp, dandruff, and erythema for minoxidil and sexual dysfunction (erectile dysfunction, low libido, anorgasmia) for oral finasteride, novel alternative AGA treatments are still needed; they continue to be a key focus of modern dermatological research [1, 8].

Several plant extracts, such as saw palmetto, white tea extract, and plant‐based oils, have been reported to potentially enhance hair growth and improve hair quality. Among these, caffeine stands out as the most notable plant‐based active ingredient, with the most extensively researched background against male and female pattern hair loss [8, 9, 10]. Caffeine has been shown to stimulate hair follicle growth through the inhibition of phosphodiesterase, which increases cyclic AMP levels and promotes cellular energy. It additionally counteracts the effects of dihydrotestosterone, a hormone responsible for androgenetic alopecia, thereby prolonging the anagen (growth) phase of hair follicles [11]. A previous study on a topically applied rinse‐off formulation has already demonstrated that caffeine effectively penetrates the skin and into the hair follicle within a short application time of just 2 min [12].

N,N‐Dimethylglycine (DMG) is a naturally occurring glycine derivative and part of the endogenous homocysteine pathway [13, 14]. DMG and its sodium salt are used as food supplements in animal husbandry and are safe nonfuel food supplements for human application [15, 16, 17, 18, 19, 20, 21, 22, 23, 24]. Previous studies have demonstrated that dimethylglycine sodium salt (DMG‐Na salt) has positive effects on crucial parameters for skin biology. Here, DMG‐Na was shown to promote cellular proliferation and migration, whereas also inducing the synthesis and release of growth factors, including vascular endothelial growth factor (VEGF), in cultured human epidermal keratinocytes. In addition, DMG‐Na treatment has been demonstrated to not only decrease inflammation and oxidative stress in various in vitro skin disease models but also to increase the influx of the signaling molecule calcium, further highlighting its potential benefits for skin health [25]. As VEGF and calcium are important regulators for the vascular system, further research has been conducted to unravel the effects of DMG‐Na on vascular endothelial functions and human skin microcirculation. In these experiments with primary human dermal microvascular endothelial cells, DMG‐Na was proven to increase (i) calcium influx, (ii) the expression and activation of endothelial nitric oxide synthase (eNOS), as well as (iii) the production of nitric oxide (NO), the master regulator of endothelial‐derived vasodilation [26]. Further clinical data revealed that a single topical application of a DMG‐Na‐containing gel led to an increase in skin microcirculation in both male and female subjects, as compared to placebo and untreated control. These findings not only highlight the physiological effect of DMG‐Na but also show its effective dermal penetration, which is a prerequisite for the observed increase in microcirculation. Given the critical role of an adequate blood supply in supporting the rapid proliferation of hair follicles, the aim of this study was to evaluate the efficacy of the combined treatment pathways of DMG‐Na and caffeine in targeting male pattern hair loss.

To date, no published studies have documented the beneficial effects of topically applied dimethylglycine sodium salt (DMG‐Na) on male pattern hair loss in vivo. In this study, we present findings from a 24‐week, randomized, double‐blind, placebo‐controlled, parallel‐group clinical trial investigating the efficacy of a shampoo containing DMG‐Na and caffeine against male pattern hair loss.

## 2 Materials and Methods

### 2.1 Ethical Conduct of the Study

This trial was conducted in compliance with the ethical principles outlined in the Declaration of Helsinki and its subsequent amendments. It adhered to international guidelines for Good Clinical Practice, specifically the ICH E6(R1) guideline dated 10/06/1996 (CMP/ICH/135/95), as well as Directive 2001/20/EC of the European Parliament and Council (published in OJ/EC on 01/05/2001). Additionally, the trial followed the Colipa guidelines from August 1997 titled “Guidelines for the Assessment of Human Skin Compatibility.” Data handling procedures were aligned with European Directive 95/46/EC concerning the protection of personal data and the free movement of such data. Prior to participation, all subjects provided written informed consent. The study protocol received approval from the internal revision committee (Opinion No. 84, dated 24/09/2021).

### 2.2 Study Design and Study Products

This trial was performed according to a randomized, double‐blind, placebo‐controlled, pre‐post parallel group study design after a washout phase (use of a basic shampoo for 10 days at home). The active shampoo (containing 1% Caffeine, 1% Sodium dimethylglycinate, and further components; Aqua, Sodium laureth sulfate, Laureth‐2, Disodium laureth sulfosuccinate, Citric acid, Sodium lauryl glutamate, Panthenol, Sodium chloride, Parfum, PEG‐120 methyl glucose dioleate, Hydrolyzed wheat protein, Sodium benzoate, Sodium citrate, Propylene glycol, Menthol, PEG‐40 hydrogenated castor oil, Potassium sorbate, Polyquaternium‐7, Disodium EDTA, Zinc PCA, Niacinamide, Phenoxyethanol, CI 42090) was tested against a shampoo base with identical conditioning performance excluding the bio‐actives. The neutral packaging of the two shampoos was identical. The samples were identified by the sponsor with a consecutive number from 1 to 154 according to a predefined randomization list (created by an independent statistical expert).

The experimental setup was designed to mimic typical home‐use conditions for shampoo application, including the area of application, the amount of product used, as well as the frequency and duration of use. Over a period of six consecutive months, participants applied 7 mL of the test shampoo daily to wet hair and scalp, allowed it to remain for 2 min after lathering, and subsequently rinsed it off.

### 2.3 Study Population

A total of 154 healthy male participants experiencing hair thinning or male pattern hair loss were enrolled in the study, with 77 individuals assigned to the active group and 77 to the placebo group. All subjects had Fitzpatrick skin phototypes II, III or IV and exhibited early signs of hair thinning and/or androgenetic alopecia corresponding to Hamilton‐Norwood classifications II to IV. Each participant had a hair count of 15–18 hairs in the “hair pull test,” conducted 2 days after the last hair wash.

In accordance with the exclusion criteria, participants had not used any hair restorer, including tablets, capsules, tonics, or shampoos, within the previous 4 weeks. Additional exclusion criteria included other types of alopecia (e.g., alopecia areata or psychosomatic forms such as trichotillomania), drug‐induced hair loss (from immunological treatments, chemotherapy, etc.), and known allergies to products similar to the investigational items (cosmetic hygiene or care products) or other potential products such as medications or foods.

### 2.4 Efficacy Evaluation

The primary efficacy endpoint was the number of hairs pulled during the hair pull test conducted after 6 months of product use [27, 28, 29]. The hair pull test was carried out by the same designated trained technician throughout the study, at the investigative site, following standardized procedures. Assessments were conducted at three time points: at Baseline after a 10‐day washout phase, but prior to any test product application (Baseline), after 3 months of continuous use (3 months), and after 6 months of treatment (6 months).

**FIGURE 1:**
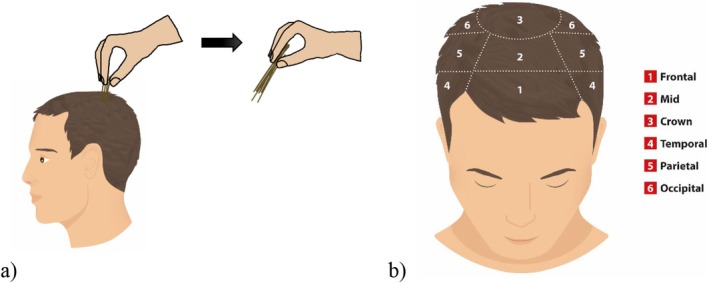
(a) Depiction of the hair pull test. (b) Overview of the different scalp areas.

For consistency, three specific scalp regions (frontotemporal, parietal and occipital) were selected for testing. Prior to the test examination, hair was left unwashed for 2 days and was neither brushed nor combed in the 2 h before the examination. In each of these areas, approximately 50 hairs were grasped together between the thumb and forefinger and gently pulled. Lost hair via pulling was collected and counted (Figure 1).

As part of the secondary efficacy assessments, participants conducted a subjective evaluation of the product's cosmetic qualities and perceived effectiveness after three and 6 months of continuous product use (see Table 1).

**TABLE 1:** Subjective assessment of the cosmetic qualities and product efficacy by the volunteer.

The product leaves your hair looking fuller/more dense (in %)	Agree	Agree somewhat	Disagree somewhat	Disagree
The product leaves your hair looking thicker (in %)	Agree	Agree somewhat	Disagree somewhat	Disagree
The product leaves your scalp less visible (in %)	Agree	Agree somewhat	Disagree somewhat	Disagree
You see less amount of hair in the comb/brush/hand (in %)	Agree	Agree somewhat	Disagree somewhat	Disagree
How do you rate the extent of hair loss? (in %)	Very slight	Slight	Moderate	Severe

A noninvasive phototrichogram analysis was conducted using the Trichoscan HD system (DermoScan, [30]) on a subgroup of 30 participants (15 out of each group) who provided written consent for scalp shaving. Equal subgroup allocation was performed by a dermatologist not involved in the measurement procedures. Assessments were carried out at Baseline, as well as after three (3 months) and six (6 months) months of continuous product use, with the objective of monitoring the total number of hairs, hair density (hair/cm^2^), percentage of hairs in the anagen phase and percentage of hairs in the telogen phase.

**FIGURE 2:**
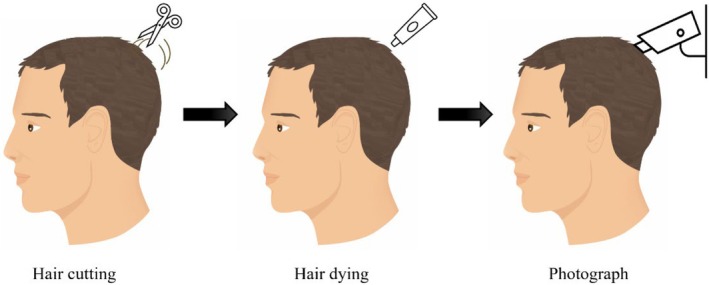
Depiction of the phototrichogram at Baseline and after treatment.

The target area for the measurement was selected within a transitional area between balding and normally growing hair of the frontotemporal or vertex region. The hair was clipped, dyed, and images were taken from the target area after 48 h (Figure 2). In order to accurately assess the identical target area after three and six consecutive months of product usage, shaving of the target area was repeated every 14 days.

### 2.5 Safety Evaluation

Local tolerance was assessed through scalp examinations carried out by the investigator at Baseline, after 3 months, and again after 6 months of product use. In addition to these clinical evaluations, tolerability was also monitored based on participant reports of any sensations of discomfort experienced during the study period.

### 2.6 Statistics

The statistical analysis of the study data was performed by an independent statistical expert. The exact Mann–Whitney Test and exact Friedman‐Test were used for primary efficacy parameters, whereas the Mann–Whitney Test, exact Fisher's Test, or Friedman‐Test was chosen for secondary efficacy parameters. The *t*‐Test was used for phototrichogram analysis. *p* values lower than 0.05 were considered statistically significant.

## 3 Results

154 men with male pattern hair loss or hair thinning were enrolled in accordance with the inclusion and exclusion criteria, each treatment arm (active and placebo) consisting of 77 subjects. The age of the subjects ranged from 18 to 65 years. Median age was 35.0 for the active group and 37.0 for the placebo group; mean age was 37.6 years for the active group and 38.2 for the placebo group, with a SD of 14.0 for both groups. The degree of subjects hair loss severity is shown in Table 2.

**TABLE 2:** Hamilton–Norwood type alopecia distribution within the study panel.

		Active		Placebo	
		*n*	%	*n*	%
Hamilton–Norwood Type II–IV	II	25	32.5	28	36.4
	II A	11	14.3	10	13.0
	III	14	18.2	8	10.4
	III A	11	14.3	10	13.0
	III vertex	10	13.0	12	15.6
	IV	6	7.8	9	11.7

### 3.1 Primary Efficacy Outcomes

**FIGURE 3:**
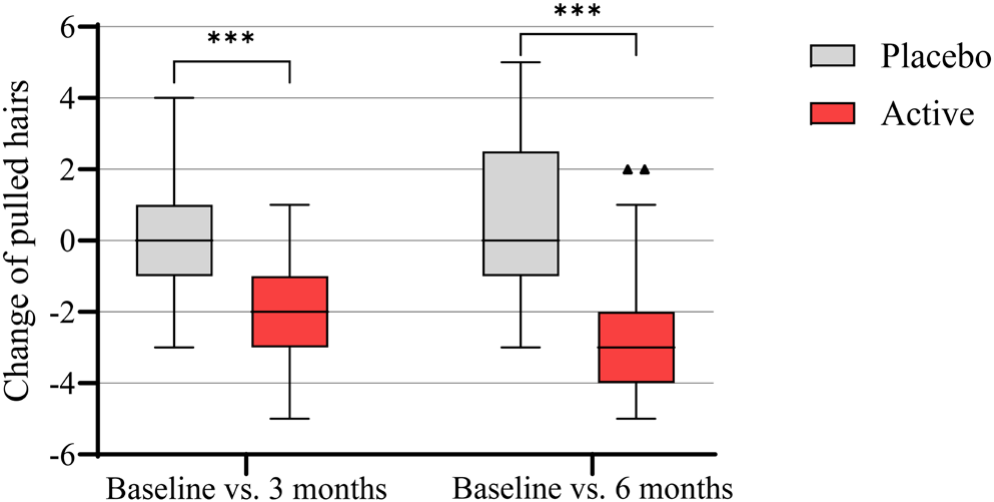
Change of pulled hairs after 3 and 6 months upon indicated treatments (active or placebo shampoo) compared to Baseline. Box and whisker plots (Tukey method) showing the median, quartiles, and extreme values for each timepoint comparison in each test group (Baseline vs. 3 or 6 months). Effects measured in the active shampoo group were compared with those measured in the corresponding placebo group, and *p* values calculated by the Mann–Whitney Test are shown as follows: ***p < 0.001 significant difference compared to placebo.

The primary efficacy endpoint was the change in the number of hairs pulled in comparison to Baseline in the hair pull test after 3 and 6 months. The results are shown in Figure 3 and Table 3:

**TABLE 3:** Descriptive statistics of pulled hairs before (Baseline), after three months (3 months), and after six months (6 months) after application of the active or placebo shampoo.

Group		Mean	SD	25% P.	Median	75% P.	Min	Max	*n*
Hair pull test Baseline	Active	16.2	1.2	15.0	16.0	17.0	15.0	18.0	77
	Placebo	16.3	1.2	15.0	16.0	18.0	15.0	18.0	77
Hair pull test 3 months	Active	14.2	1.7	13.0	14.0	15.0	10.0	17.0	77
	Placebo	16.6	2.0	15.0	17.0	18.0	12.0	22.0	77
Hair pull test 6 months	Active	13.4	1.9	12.0	13.0	15.0	10.0	19.0	77
	Placebo	16.9	2.6	15.0	17.0	19.0	13.0	22.0	77
Hair pull test Baseline vs. 3 months	Active	−1.9	1.4	−3.0	−2.0	−1.0	−5.0	1.0	77
	Placebo	0.3	1.7	−1.0	0.0	1.0	−3.0	4.0	77
Hair pull test Baseline vs. 6 months	Active	−2.8	1.6	−4.0	−3.0	−2.0	−5.0	2.0	77
	Placebo	0.6	2.2	−1.0	0.0	2.0	−3.0	5.0	77

Abbreviations: 25% P., 25% percentile; 75% P., 75% ercentil; SD, standard deviation.

After 6 months of treatment, the reduction in the number of hairs pulled via the hair pull test was significantly greater in the active shampoo group compared to the placebo group (mean change: −2.8 ± 1.6 vs. 0.6 ± 2.2 *p* < 0.001; exact Mann–Whitney Test). A similar statistically significant difference was already evident after 3 months of product use (*p* < 0.001; exact Mann–Whitney Test).

In addition, exact Friedman‐Test showed that the number of hairs pulled significantly decreased after using the active shampoo (16.2 ± 1.2 at Baseline, 13.4 ± 1.9 after 6 months; *p* < 0.001). In contrast, the placebo group showed no significant change from Baseline to 6 months (16.3 ± 1.2 at Baseline, 16.9 ± 2.6 after 6 months; *p* = 0.300).

### 3.2 Secondary Efficacy Outcomes

#### 3.2.1 Subjective Evaluation

**FIGURE 4:**
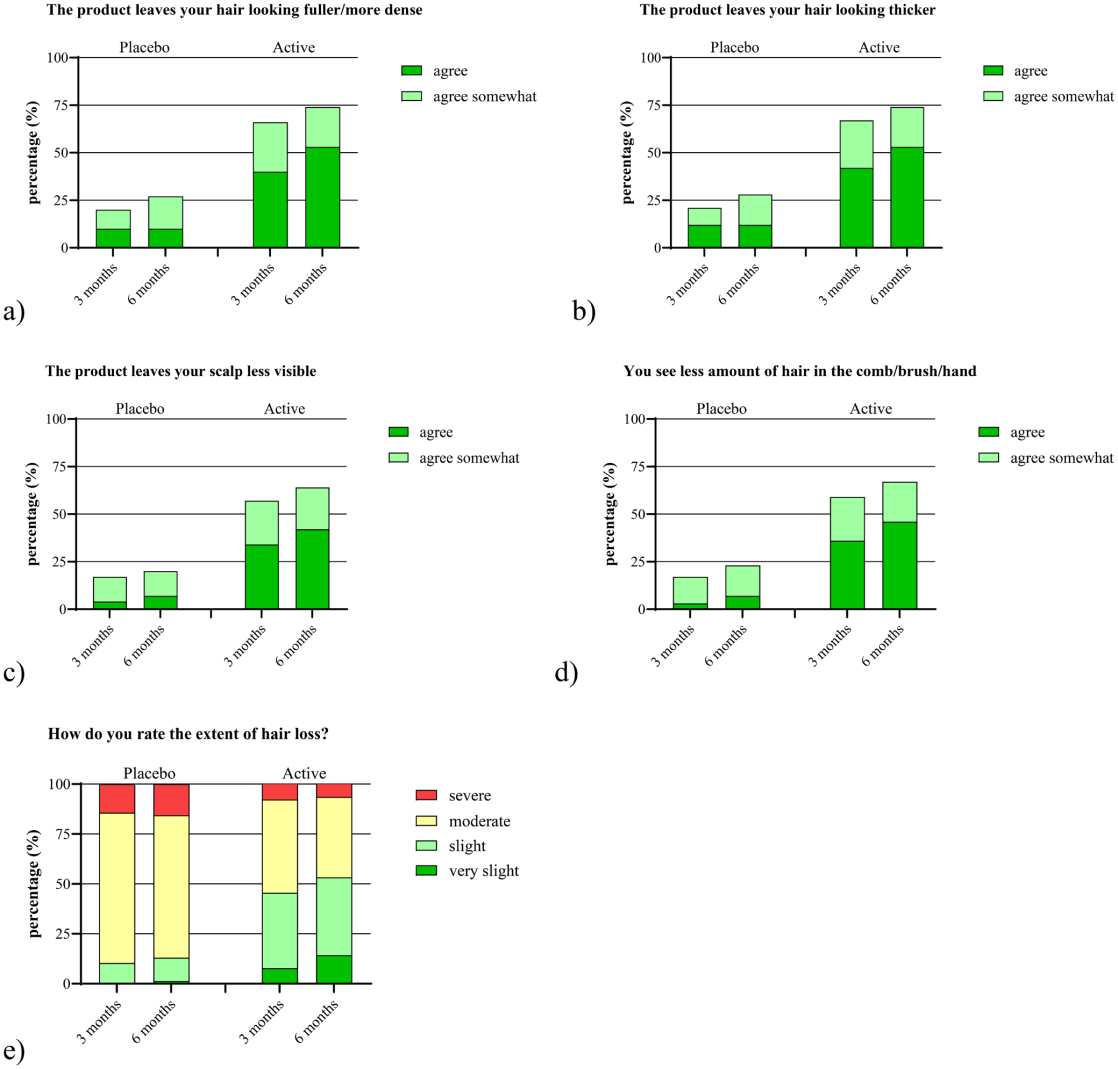
Subjective evaluation of the investigational products by the subjects after three and after 6 months, two‐sided *p* values of exact Mann–Whitney *U*‐Test (placebo vs. active shampoo after 6 months). (a) Hair density, *p* < 0.001. (b) Hair thickness, *p* < 0.001. (c) Scalp visibility, *p* < 0.001. (d) Amount of hair loss during combing, *p* < 0.001. (e) Extent of hair loss rating, *p* < 0.001.

Secondary efficacy endpoints included the participants' subjective assessment of the cosmetic properties and perceived effectiveness of the investigational products. After 6 months of use, the active shampoo was rated significantly higher than the placebo shampoo in terms of both cosmetic quality and efficacy in reducing hair loss (*p* < 0.05 for all comparisons, Mann–Whitney Test; see Figure 4).

#### 3.2.2 Phototrichogram Evaluation

**FIGURE 5:**
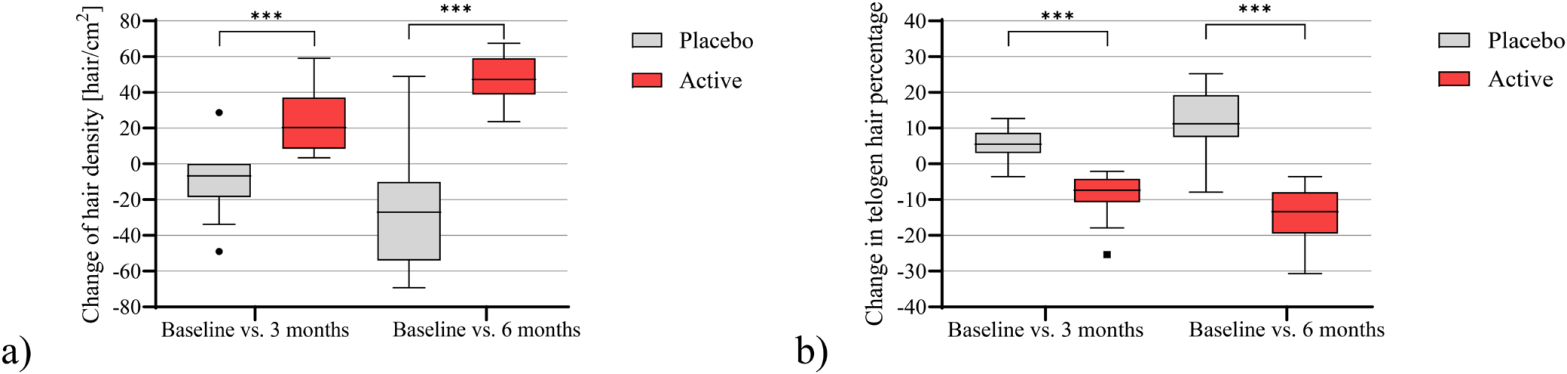
Phototrichogram analysis of hair growth parameters presented as changes compared to Baseline after 3 and 6 months upon indicated treatments (active or placebo shampoo). (a) Hair density. (b) Percentage of telogen hairs (%). Effects measured in the active shampoo group were compared with those measured in the corresponding placebo group, and *p* values calculated by *t*‐Test are shown as follows: ***p < 0.001 significant difference compared to placebo.

A phototrichogram analysis was conducted on a subgroup of 30 male participants (Table 4 and Figure 5). After 6 months of product application, the active shampoo group showed significantly greater improvements compared to the placebo group, including an increase in total hair count, hair density, and the percentage of hairs in the anagen phase, along with a corresponding decrease in the proportion of hairs in the telogen phase (*p* < 0.001, *t*‐Test).

**TABLE 4:** Descriptive statistics of the time course of TrichoScan parameters before application (Baseline), after 3 months and after 6 months application of the investigational shampoo products.

Group		Mean	SD	25% P.	Median	75% P.	Min	Max	n
Total number of hairs Baseline	Active	77.7	22.2	59.0	78.0	99.0	38.0	110.0	15
	Placebo	91.9	24.4	79.0	83.0	124.0	61.0	137.0	15
Total number of hairs 3 months	Active	92.2	26.7	74.0	96.0	101.0	42.0	140.0	15
	Placebo	85.8	28.5	68.0	79.0	109.0	40.0	137.0	15
Total number of hairs 6 months	Active	105.7	24.2	87.0	107.0	122.0	54.0	144.0	15
	Placebo	76.1	30.7	54.0	68.0	108.0	37.0	137.0	15
Hair density Baseline	Active	131.3	37.5	99.7	131.8	167.2	64.2	185.8	15
	Placebo	155.2	41.3	133.4	140.2	209.5	103.0	231.4	15
Hair density 3 months	Active	155.7	45.1	125.0	162.2	170.6	70.9	236.5	15
	Placebo	144.9	48.1	114.9	133.4	184.1	67.6	231.4	15
Hair density 6 months	Active	178.6	40.8	147.0	180.7	206.1	91.2	243.2	15
	Placebo	128.6	51.9	91.2	114.9	182.4	62.5	231.4	15
Anagen % Baseline	Active	61.4	7.5	52.8	62.9	67.1	49.6	74.1	15
	Placebo	70.9	5.9	66.1	70.6	76.0	61.2	79.6	15
Anagen % 3 months	Active	70.0	7.1	63.7	70.3	75.5	55.9	81.5	15
	Placebo	65.5	6.8	59.8	66.3	72.0	53.4	76.3	15
Anagen % 6 months	Active	75.7	5.5	70.6	75.0	79.3	68.3	85.7	15
	Placebo	58.7	9.8	54.7	57.6	65.2	41.7	76.3	15
Telogen % Baseline	Active	38.6	7.5	32.9	37.1	47.2	25.9	50.4	15
	Placebo	29.1	5.9	24.0	29.4	33.9	20.4	38.8	15
Telogen % 3 months	Active	30.0	7.1	24.5	29.7	36.3	18.5	44.1	15
	Placebo	34.5	6.8	28.0	33.7	40.2	23.7	46.6	15
Telogen % 6 months	Active	24.3	5.5	20.7	25.0	29.4	14.3	31.7	15
	Placebo	41.2	9.5	34.8	42.4	45.3	23.7	58.3	15

The descriptive statistics of the assessed phototrichogram parameters after 3 and 6 months compared to Baseline are presented in Table 5.

**TABLE 5:** Change of TrichoScan parameters after three months and six months of investigational shampoo product use compared to the Baseline (*p* values, results of two‐sided *t*‐Test for independent groups).

Group		Mean	SD	25% P.	Median	75% P.	Min	Max	*n*	*p*
Number of hairs Baseline vs. 3 months	Active	14.5	10.3	5.0	12.0	22.0	2.0	35.0	15	< 0.001
	Placebo	−6.1	10.7	−11.0	−4.0	0.0	−29.0	17.0	15	
Number of hairs Baseline vs. 6 months	Active	28.0	7.7	23.0	28.0	35.0	14.0	40.0	15	< 0.001
	Placebo	−15.7	17.7	−32.0	−16.0	−6.0	−41.0	29.0	15	
Hair Density Baseline vs. 3 months	Active	24.4	17.4	8.5	20.3	37.1	3.4	59.1	15	< 0.001
	Placebo	−10.2	18.1	−18.6	−6.7	0.0	−49.0	28.7	15	
Hair Density Baseline vs. 6 months	Active	47.3	13.0	38.9	47.3	59.1	23.7	67.5	15	< 0.001
	Placebo	−26.6	29.9	−54.1	−27.0	−10.1	−69.3	49.0	15	
Anagen % Baseline vs. 3 months	Active	8.6	6.1	4.2	7.4	10.7	2.1	25.4	15	< 0.001
	Placebo	−5.3	4.4	−8.7	−5.5	−3.0	−12.7	3.6	15	
Anagen % Baseline vs. 6 months	Active	14.3	7.3	7.9	13.4	19.5	3.6	30.7	15	< 0.001
	Placebo	−12.2	9.0	−19.2	−11.2	−7.5	−25.2	7.9	15	
Telogen % Baseline vs. 3 months	Active	−8.6	6.1	−10.7	−7.4	−4.2	−25.4	−2.1	15	< 0.001
	Placebo	5.3	4.4	3.0	5.5	8.7	−3.6	12.7	15	
Telogen % Baseline vs. 6 months	Active	−14.3	7.3	−19.5	−13.4	−7.9	−30.7	−3.6	15	< 0.001
	Placebo	12.1	8.8	7.5	11.2	19.2	−7.9	25.2	15	

Further within‐group analysis using exact Friedman‐Test revealed a significant increase in hair density after 6 months of active shampoo usage (*p* < 0.001), whereas hair density significantly decreased after the use of the placebo shampoo (*p* < 0.001). The percentage of telogen hairs (%) significantly decreased after using the active shampoo for 6 months (*p* < 0.001; exact Friedman‐Test), whereas the percentage of telogen hairs (%) significantly increased after the use of the placebo shampoo (*p* < 0.001; exact Friedman‐Test).

**FIGURE 6:**
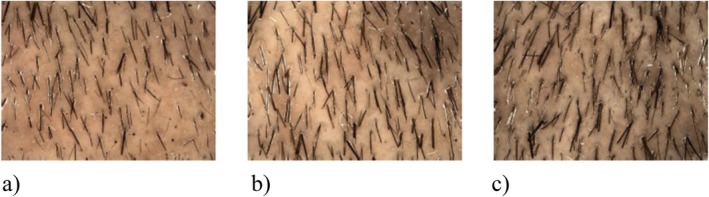
Photo documentation of hair growth at one scalp site at (a) Baseline, (b) after 3 months and (c) after 6 months of active shampoo application.

Figure 6 shows the photographically visualized progress of hair growth during phototrichogram analysis of one scalp site for one subject at Baseline, after 3 months, and after 6 months of daily application of the active shampoo.

### 3.3 Adverse Events

No adverse events were observed during the entire study period. Both the active and placebo shampoos were well tolerated and considered to have very good skin compatibility.

## 4 Discussion

This is the first clinical trial to demonstrate the safety and efficacy of topically applied dimethylglycine sodium salt (DMG‐Na salt) in combination with caffeine against male pattern hair loss.

Dimethylglycine, as demonstrated very recently, upregulates the expression and synthesis of important regulators for endothelial‐derived vasodilation, leading to increased blood circulation in the skin. The synthesis of nitric oxide (NO), the primary regulator for maintaining vascular homeostasis, as well as the expression of the NO‐producing enzyme eNOS, which increases significantly with augmented calcium concentrations, was significantly upregulated upon DMG‐Na treatment in human dermal microvascular endothelial cells [26]. As dermal blood supply is crucial for the highly metabolically active hair follicle cells, by providing nutrients, growth factors, cytokines, and other molecules but also by removing waste products from the hair follicle, circulatory support by DMG‐Na during the anagen phase is an important target to support hair growth and reduce hair loss. Early studies in the 1960s already revealed that blood supply is abundant during the early anagen phase and sparse during the telogen phase. Both age and the occurrence of pattern hair loss were shown to diminish hair follicle blood supply, both in male and female subjects [31, 32, 33].

Interestingly, Minoxidil, first introduced as a vasodilator for treating hypertension in the early 1970s and later approved for treating hair loss, was also shown to not only induce microcirculation but also induce the expression and release of similar factors, including, that is, calcium and VEGF [34, 35].

We acknowledge certain limitations of this clinical study, including a limited number of subjects undergoing phototrichogram analysis, the absence of standardized global photography, and a limited follow‐up duration. Nevertheless, all efficacy assessments consistently demonstrated a clear superiority of the treatment compared to the placebo. After the 24‐week study period, the number of pulled hairs via hair pull test was significantly reduced upon daily application of the DMG‐Na and caffeine‐containing shampoo product. The hair pull test was conducted by the same trained technician, ensuring the highest standard conditions for this hair loss measurement. Although a direct statistical comparison with prior studies on caffeine‐based shampoos is precluded by variations in study protocols and efficacy assessments, the observed outcomes indicate a superior efficacy associated with the combined application of caffeine and DMG‐Na [36, 37]. This enhanced efficacy is further supported by the subjects' own evaluations, which revealed significantly better ratings for the DMG‐Na and caffeine‐containing shampoo compared to the placebo, particularly in terms of cosmetic quality and perceived effectiveness. Several questions addressing the perception of hair, scalp, and hair loss status showed significantly better results after three and 6 months of active shampoo treatment. Despite the comparatively low sample size for the phototrichogram analysis, also the total number of hairs, the hair density, the percentage of anagen hairs, and the percentage of telogen hairs were statistically significantly improved after application of the active shampoo compared to the use of the placebo shampoo. Although the percentage of anagen hairs in this study, ranging from 60% to 70% at Baseline, appears to be relatively low compared to typically reported anagen to telogen ratios of approximately 5:1 for androgenetic alopecia, similar values have been observed in prior clinical trials [38, 39, 40]. These trials particularly measured hair density at the leading edge of the vertex balding scalp, a measurement site comparable to the one used in this study. Taken together, these efficacy data demonstrate the high potential for this effective and safe combinational treatment against male pattern hair loss.

## 5 Conclusion

This innovative dimethylglycine sodium salt and caffeine‐containing shampoo proved to be a safe and effective treatment for male pattern hair loss; significantly outperforming a corresponding placebo in reducing pattern hair loss and improving hair growth parameters, such as hair density and the anagen/telogen ratio.

## Author Contributions

All authors have read and approved the final version of the manuscript.

## Conflicts of Interest

M.B. and J.M.V. are employees of Dr. Kurt Wolff GmbH & Co. KG, Bielefeld, Germany. E.S.Z.W. is an employee of Dr. August Wolff GmbH & Co. KG Arzneimittel, Bielefeld, Germany.

## Data Availability

The data that support the findings of this study are available from the corresponding author upon reasonable request.
